# Environmental and health implications of Pb-bearing particles in settled urban dust from an arid city affected by Pb–Zn factory emissions

**DOI:** 10.1038/s41598-023-48593-5

**Published:** 2023-12-02

**Authors:** M. F. Soto-Jiménez, S. Roos-Muñoz, S. Soto-Morales, L. E. Gómez-Lizarrága, L. Bucio-Galindo

**Affiliations:** 1 Unidad Académica Mazatlán, Instituto de Ciencias del Mar y Limnología, Universidad Nacional Autónoma de México, Mazatlán, Sinaloa México; 2 Tecnológico Nacional de México/Instituto Tecnológico de Mazatlán, Mazatlán, Sinaloa México; 3 Instituto Nacional de Salud Pública, Cuernavaca, Morelos México; 4 Instituto de Ciencias del Mar y Limnología, Universidad Nacional Autónoma de México, Ciudad Universitaria, Mexico City, México; 5 Laboratorio de Cristalografía y Materiales Naturales, Instituto de Física, Universidad Nacional Autónoma de México, Ciudad Universitaria, Mexico City, México

**Keywords:** Environmental chemistry, Environmental impact, Environmental sciences

## Abstract

Metal-rich particles originating from non-ferrous metallurgical activities are the primary source of atmospheric metals in urban environments. These particles vary in size, morphology, and elemental compositions and they undergo weathering processes that alter their composition and affect their toxicity. This study focuses on lead (Pb)-rich particles in settled urban dust within an arid and dusty city, Torreón in North Mexico, affected by Met–Mex Peñoles complex, one of the world's largest Ag–Cd–Pb–Zn smelting and refining facilities in operating since 1901. Torreón is characterized by arid conditions, temperature fluctuations, and low humidity. Dry atmospheric particles were collected in 2015 and 2017 from Torreón's urban area within a 3 km radius of the Met–Mex Peñoles complex. We used various analytical techniques, including scanning electron microscopy (SEM), Energy Dispersive X-ray Spectroscopy (EDS), and X-ray powder diffraction (XRD) to determine the size, morphology, elemental composition and mineralogy of Pb-bearing particles. Our analysis revealed a range of Pb-bearing particle sizes and morphologies with varying Pb (0.3 to 51–87.2%) and other element contents, such as As (0.04 to 1–3.4%), Cd (0.4 to 3.3–5.1%), Cu (0.51–14.1%), Hg (ND-0.6%), and Zn (1.7 to 79–90.3%). XRD analysis confirmed the presence of Pb and Zn sulfides, Pb carbonates, Pb sulfate, and Pb oxides in urban dust, both as individual particles and agglomerates. Primary Pb minerals were linked to fugitive feed concentrates and smelter flue gas at Met–Mex Peñoles, while secondary Pb minerals, like Pb carbonates, Pb sulfate, and Pb oxides, resulted from direct emissions and weathering processes. Compared to galena, secondary Pb minerals exhibit higher chemical availability in the environment, posing greater risks to the environment and human health. As the particles analyzed are presumed to be resuspended rather than freshly emitted by Met–Mex, the presence of secondary Pb minerals in settled urban dust is predominantly linked to weathering processes. The physical and chemical transformations in Pb-rich particles contribute to increased Pb bioavailability and toxicity in urban dust, with substantial implications for environmental and human health. These findings highlight the potential consequences of weathered Pb-rich particle in urban areas, particularly in the arid and dusty city of Torreón.

## Introduction

Fugitive and non-controlled emissions of metal-rich particles from mining and non-ferrous metallurgical are the main source of metals of atmospheric metals^1,2^, which in turn can affect urban environments, biota, and humans^3–5^. The physical and chemical properties of these metal-rich particles in urban impacted by mining and metallurgical activities depend on the type of processed mineral, industrial stage, and the emission controls in place. In the context of smelting and refining of silver (Ag), cadmium (Cd), lead (Pb), and zinc (Zn), the process results in the emission of metal-rich particles into the atmosphere. These particles exhibit various characteristics, including varying sizes, diverse morphologies, and elemental and mineral compositions^6–11^. These particles contain high levels of potentially toxic elements (PTEs), such as arsenic (As), Cd, copper (Cu), mercury (Hg), manganese (Mn), Pb, and Zn.

While metal-rich particles in urban environments, such as those found in urban soils, dust, and aerosols, are typically relatively stable, they undergo gradual weathering processes that alter their composition and can significantly affect metal availability and toxicity. Metal-rich particles emitted from smelter and refineries complexes, as well as slags, undergo significant physical and chemical transformations throughout various stages, including atmospheric emission, precipitation, and subsequent accumulation in topsoil and urban dust^11–14^. The extent of these weathering processes on Pb slags and metal-rich particles within urban areas depends on prevailing deposition conditions, including oxidizing conditions, water content, temperature, pH, microorganisms, and presence of other inorganic and organic compounds^15–18^.

One of the largest Ag–Cd–Pb–Zn smelting and refining complexes in the world, Met–Mex Peñoles, is in Torreón, a dusty and arid city in northern Mexico. The complex has been in operation since 1901 and has been a significant source of metal-rich particles and PTEs in both rural and urban areas of Torreón for decades^4,5,19–22^. Soto-Jimenez and Flegal^6^ estimated that the complex has emitted between 23,350 and 27,580 tons of Pb-rich particles over the past 120 years, with the largest emissions (63–75% of total emissions) occurring during the pre-1960 period when fugitive and emission controls were negligible.

The impact of weathering on the physicochemical properties of Pb-rich smelting particles that have been emitted for over a century has not yet been studied in Torreón. The weathering process on metal rich-particles can influence the mobility and availability of metals and exacerbate their potentially harmful effects on human health and the environment. However, little is known about these mechanisms in arid climate regions^23^. The kinetic weathering process of Pb-rich particles in arid climates may be unique due to the significant temperature fluctuations and scarce humidity in these environments.

In this research, we conducted a comprehensive analysis of dry deposited dust samples collected from urban areas surrounding the Met–Mex Peñoles complex. Our investigation aimed to characterize metal-rich particles within the dust collected from Torreón's urban environment. We employed advanced techniques, including scanning electron microscopy (SEM) to visualize the particles and Energy Dispersive X-ray Spectroscopy (EDS) to determine their elemental composition through electron beam bombardment. Furthermore, we focused our attention on lead (Pb)-rich particles, utilizing SEM–EDS and X-ray powder diffraction (XRD) methods to examine their size distribution, morphology, as well as their elemental and mineral composition.

The primary objective of this study was to gain insight into the physical and chemical transformations experienced by Pb-rich particles emitted from one of the world's largest Ag–Cd–Pb–Zn smelting and refining complexes. We conducted this analysis within the urban environment of Torreón, characterized by severe arid conditions. These investigations are pivotal as the physical and chemical properties of Pb-rich particles under such conditions play a critical role in determining the bioavailability and toxicity of lead. These factors carry substantial implications for both the urban environment and human health.

## Methods

### Study area description

Torreón is a typical arid and dusty city located in northern México, with a population of over 725,000^24^. It is the largest of four sister cities of the metropolitan “La Laguna” zone (Torreón and Matamoros in Coahuila, and Gómez-Palacios and Lerdo in Durango), with a combined population of 1.8 million inhabitants^24^.

The “La Laguna” region is characterized by an arid to semiarid climate, with very low precipitation (mean 225 mm year^−1^), elevated evaporation (mean 2000 mm year^−1^), and average summer and winter temperatures of 31 and 16 °C, respectively^25^. The relative humidity average is 38%. Based on a 10-year wind vector analysis, the prevalent wind in the region blows from northwest to southeast during winter and from northeast to southwest for the rest of the year. Thermic inversions are common from November to March, exacerbating ambient pollution in the city because the pollutants are trapped in the atmospheric upper layers. Powerful convection flows are generated due to contact between compressed cold air and warm air, which can lead to resuspension and mobilization of particles into the atmosphere. The resuspension may result in dust storms (called “tolvaneras” in the region) that emit large amounts of dust into the atmosphere, with the potential to be transported over long distances. Tolvaneras, or dust storms, occur around 8–12 times in the region, with an average wind velocity of 19.4 m s^−1^^26^. Most common direction of tolvaneras is northeast and eastward of the city^27^.

The region is situated at an elevation of 1130 m above sea level (masl) and is characterized by a North–South-oriented mountain chain known as the Sierra de las Noas, which reaches a height of over 400 m (1503–1574 masl). The flat basin of the Agua Naval River divides the cities of Gomez and Lerdo and then flows southward towards Torreón City.

### Sample collection

**Figure 1:**
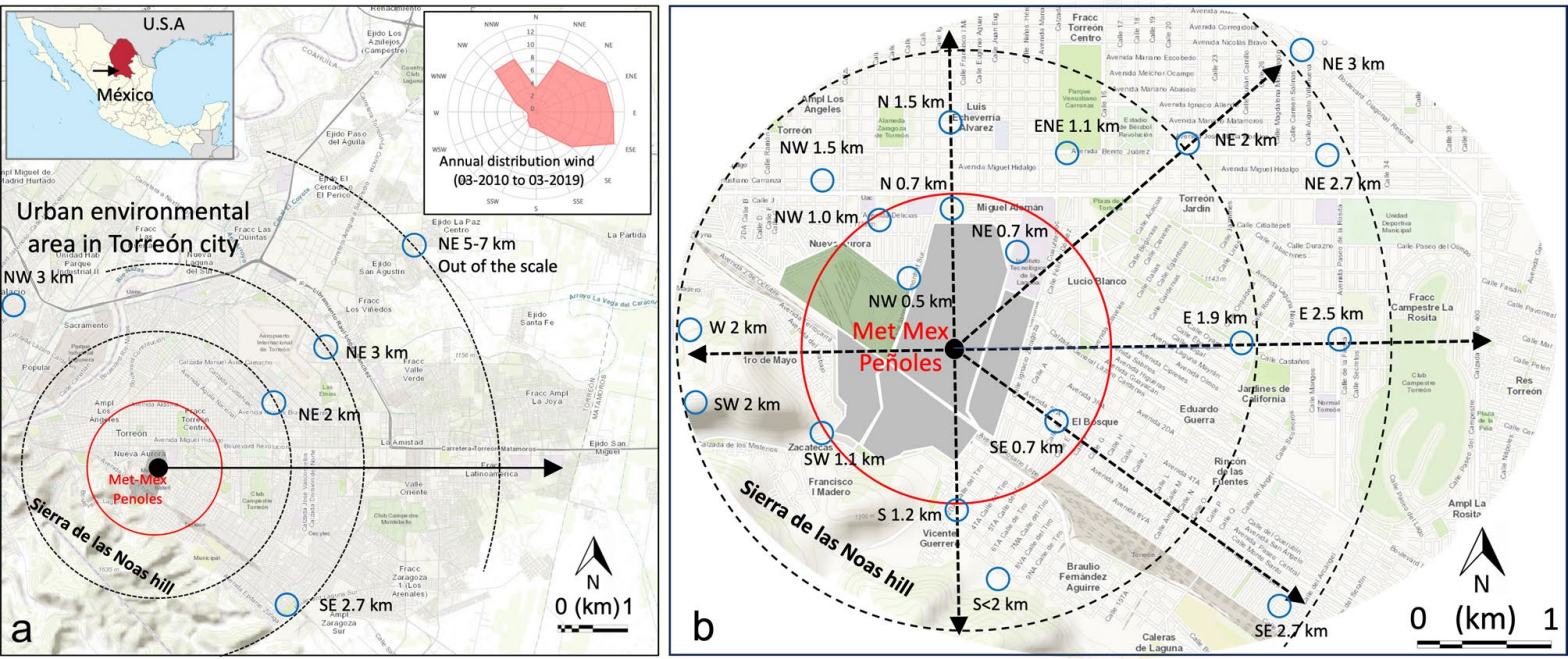
(**a**) Map of Torreón city (northern México), annual distribution wind (2010–2019), localization of Met–Mex metallurgical complex, and urban dust fall collection sites. (**b**) Geographical depiction of collection sites within the urban environment of Torreón city, extending to a radius of 2 km from the Met–Mex complex. Blue circles represent sites in the vicinity of Met–Mex, and black circles represent distant sites. Map modified from EnviroAtlas^62^.

Dry deposition of atmospheric particulate matter (urban dust) was collected in the urban area of Torreón at cross-sectional sites, selected based on measured wind direction and distance from the Met–Mex complex (Fig. 1a). Collection was conducted using eight acrylic frame plates, each measuring 1 m^2^ with a depth of 0.3 m, covered with low-density polyethylene (LDPE) as deposition devices for dry particle collection^4^. Each collection device was placed on rooftops of houses (> 3 m height), to minimize direct influence from turbulence generated by road transportation and wind, for a period of 12–48 h. The LDPE surface was cleaned using a 2 M HCl solution and disposable towelettes and sprayed with concentrated HNO_3_ (dust sticky tray). Deposited urban dust was subsequently collected using GosthWipes™ sprayed with Triton X-100 (a nonionic surfactant, Fisher Scientific Inc., NJ, USA). To compute the detection limit (LOD) and limit of quantification (LOQ) of the dust deposition fluxes, ten wipes were used as field blanks. The deposition devices were placed in a total of 12 cross-sectional sites aligned depending on the measured wind direction. These sites were located along a 6-km-transect parallel to the Sierra de las Noas hill and within a 3 km radius around the primary smelter stack of the Met–Mex complex (Fig. 1b). In addition to these sites, urban dust samples were also collected at three other locations that were far away from the complex emissions or any other known source of lead, and these sites were considered representative of the urban background in Torreón. The background composite samples were collected at distances of 4–7.2 km to the north–northeast of the Met–Mex complex. Five sampling surveys, each lasting for 5 days, were conducted during the winter–spring (January–April 2015) and spring–summer (April–May 2017) seasons to capture the prevailing weather conditions in the region. A total of 148 urban dust samples were collected. The settled urban dust collected in our study are presumed to be resuspended rather than freshly emitted by Met–Mex.

### Sample processing and analyses

All atmospheric particulate matter samples were processed and analyzed in HEPA-filtered air (Class 1000), using high-purity reagents of trace metal grade and water (18 MΩ cm (Academic Milli-Q). The processing and analysis were performed in clean trace metal laboratory located at UNAM in Mazatlán, México. To prepare the GhostWipes, they were first dried for 72 h at 65–70 °C, then equilibrated in a desiccator for 48 h before being weighed with a Sartorius semi-micro balance at 50% humidity and a temperature of 22 ± 1 °C. The dust deposition flux (g of dust m^−2^ day^−1^) was estimated using the weight difference between the short-term sample and the average GhostWipes™ blank. Both were dried to a constant weight and divided by the sampling time.

### Scanning electron microscope and Energy Dispersive X-ray Spectroscopy analyses

To determine the size and morphology and for the observation of topographic structures of the particles of the deposited urban dust samples, we analyzed 32 selected samples using scanning electron microscope (SEM). In our research, the JEOL JSM6360LV SEM was operated under high vacuum with a beam scan accessory V (20 kV) to capture electron images across a wide magnification range (from 3000× to 1,000,000×). JEOL JSM6360LV SEM is equipped with secondary and backscattered electron (BSE) detectors. Elements with a high atomic number, such as Pb, produces intense bright white color due to the emission of more backscattered electrons when the primary electron beam interacts with the nuclei of the element.

We prepared a few milligrams of urban dust samples by sprinkling aliquots onto a carbon-impregnated tape and mounting them in aluminum holders. The high-resolution SEM images (ranging from 640 × 480 to 5120 × 3840) were analyzed using software-assisted methods (ImageJ software, developed by the US National Institutes of Health) to determine the diameter of particles. SEM provided magnified image to study the size, morphology, and shape of around 40,700 individual dust particles (760–1235 particles per sample).

Particles were classified into five different size fractions based on their deposition efficiency along the respiratory tract^28–30^. Briefly, (1) particles > 10 μm were considered inhalable since they can easily enter the nose and mouth, (2) particles < 10 μm (PM_10_) were considered thoracic since they can penetrate deep into the respiratory system and generally deposited by impaction in the extrathoracic airway region, (3) particles < 4 μm were considered respirable because they are small enough to pass completely through the respiratory system and enter the bloodstream and are deposited by impaction in the tracheobronchial airway in the deep lungs, (4) particles < 2.5 μm (PM_2.5_) were considered fine because they penetrate the smaller airways and are deposited by sedimentation in the peripheral lung region in the tracheobronchial airway close to alveolar interstitium, and (5) particles < 0.1 μm (PM_0.1_) were considered ultrafine particles because they penetrate the smaller airways and are deposited by diffusion, often in the alveolar interstitium. Inhalable particles were further divided into two categories: large particles (> 10–25 μm), and giant particles (> 25 μm).

Scanning Electron Microscope is also equipped with Energy Dispersive X-ray Spectroscopy (EDS) that allow for the acquisition of both topographic images and the chemical composition of individual particles as a function of their size within settled dust samples. Our SEM–EDS configuration offers a resolution of approximately 1 nm, with an adjustable spot size and an accelerating voltage ranging from 0 to 30 kV. The EDS technique primarily relies on differences in atomic number (Z) to identify and quantify elements. When two elements have closely spaced atomic numbers, such as Pb (Z = 82) and S (Z = 16), it can be challenging to differentiate them effectively. Our detector was calibrated to characterize the X-ray peaks of individual and combined elements using the commons standards (e.g., SiO_2_, MgO, Al_2_O_3_, K-feldspar, FeS_2_, and metallic Pb, Zn, Cd, Fe, Ni, and Cu). Considering the overlap between the Pb Mα (2.34 keV) and S Kα (2.31 keV) lines, the detector was also calibrated to characterize the X-ray peaks of individual and combined Pb and S on the spectrum using crocoite (Pb-Mα) and anhydrite (S-Kα). Moreover, the EDS equipment software includes deconvolution algorithms that quantify the combinations of Pb and S in the settled urban samples. To further validate the elemental composition of the identified minerals, we conducted an additional analysis using artificially enriched dust standards with varying weight percentages of typical Pb minerals. Aliquots of Clean Loam Soil (SUPELCO CLNLOAM6) were spiked with known quantities of homogeneous lead (Pb) minerals, including galena (PbS), litharge (PbO), cerussite (PbCO_3_), and anglesite (PbSO_4_). These minerals are commonly encountered in environments affected by lead–zinc smelting and refining activities, where Pb contamination is prevalent^14,31–34^. The urban dust samples and artificial enriched standards were prepared for SEM–EDS analysis following the procedure of Bern et al.^35^. A sample aliquot (0.1–0.2 g) was weighted, and a sample mass between 10 and 20 mg was obtained by splitting. The sample was then suspended in isopropanol (10 mg dust mL^−1^), transferred to a polycarbonate substrate (0.4 µm pore size filters affixed to 13 mm aluminum stubs with conductive carbon adhesive tabs), air-dried, and coated with a thin (∼ 100 Å) conductive carbon film using a carbon evaporator with a rotating stage. EDS spectrums were collected for 200 s at a beam energy of 20 keV, probe current of 100 pA, count rate approximately 1000 cps, and working distance of 23 mm (Full Scale: 20 keV (20 eV/ch, 1 Kch), Acc. Volt: 20.0 kV, Probe Current: 1.781E−08 A). Detection limit (wt. %) for elements were between 0.05 and 0.1%, while the elemental compositions showed accuracies and precisions close to 1%.

The Pb-bearing particles in selected urban dust samples (with weathered Pb-rich particles) and artificially enriched soils were subsequently analyzed using an X-ray powder diffraction analysis coupled with SEM to identify the Pb phases^36^. XRD measurements were performed using a Philips PW 1729 X-ray diffractometer, adapted to the SEM, and operated at a voltage of 40 kV and a current of 30 mA, with Co Kα (1.79 Å) and Cu Kα radiation (1.54 Å), respectively. Diffraction patterns from the Pb-rich particles were collected over a scan range of 5–75° 2θ, with a step size of 0.02° 2θ and dwell times of 4–10 s.

### Statistical analysis

To evaluate the analytical data, univariate, bivariate and multivariate analysis, and principal component analysis. We first conducted basic descriptive statistics (e.g., median, 10th–90th, average, SD, and minimum and maximum) on the metal measurements obtained from urban dust samples. We evaluated differences between urban areas categorized by distance and direction of the Met–Mex, while accounting for season. Because the assumptions of normality and homogeneity of variance were not met and the number of samples in each distance-direction was different, we used the non-parametric Kruskal–Wallis test to evaluate the differences between groups. If the Kruskal–Wallis test detected a significant difference, we used Dunn’s post-hoc test to adjust the p-values for multiple comparisons.

## Results

### Dust deposition fluxes

**Figure 2:**
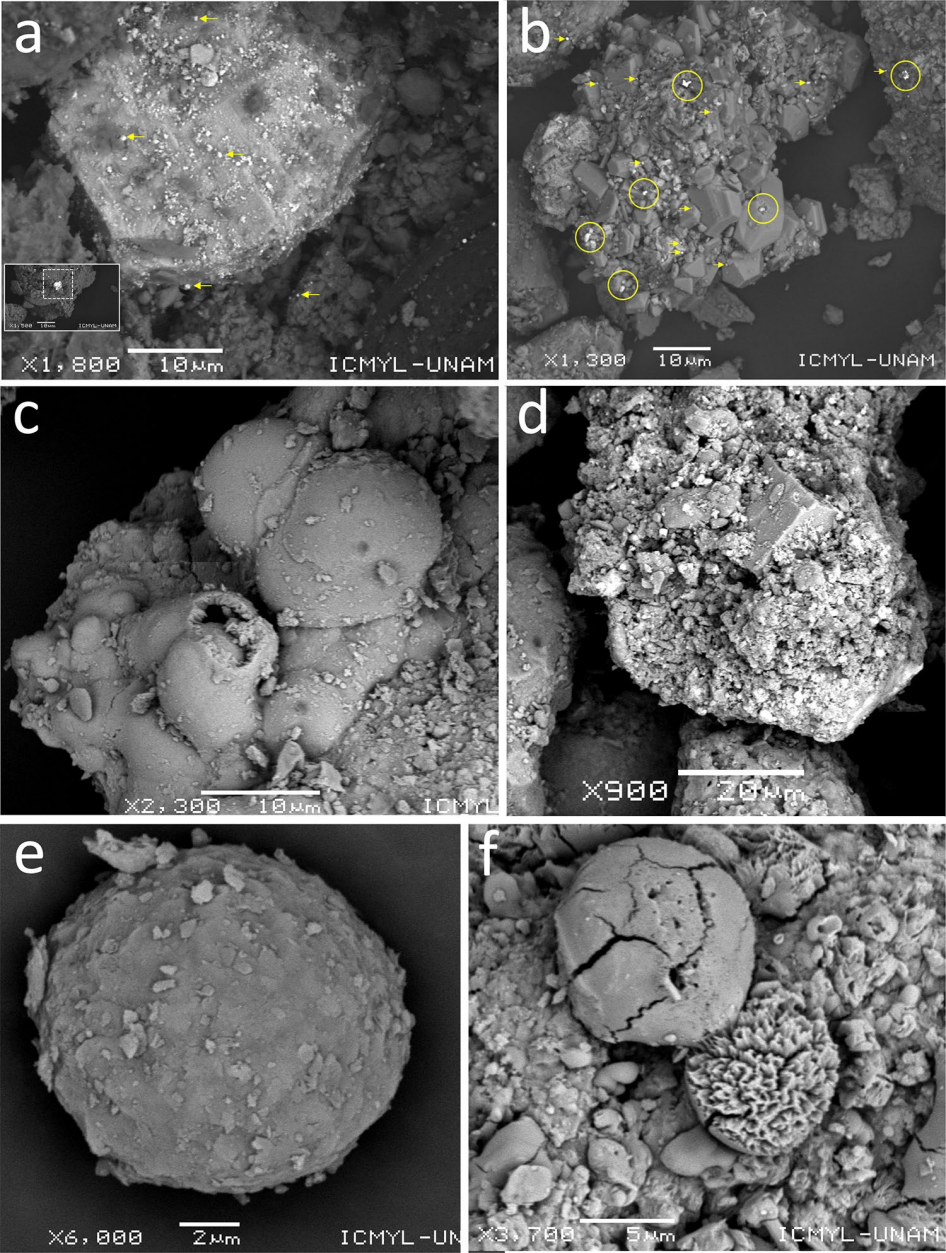
Micromorphology of metal-rich particles in urban dust observed by SEM: (**a**) small Pb-rich particles adhered to coarse smelter slag, (**b**) small Pb-rich particles incorporated to unaltered sulfides from concentrate ores, (**c**) metal-rich agglomerates particle, (**d**) galena incorporated into metal-rich agglomerates particle, (**e**) small spherical metal-rich particle, (**f**) spherical metal-particles into metal-rich agglomerates slag.

The dust deposition fluxes in Torreón during the 2015 and 2017 surveys are shown in Table S1 and Fig. S1. Significant spatial variability was observed in the deposition fluxes of atmospheric particulate matter (minimum < 0.1 to maximum of 1.7 g m^−2^ day^−1^) in Torreón, with highest averages at locations < 1 km southwest (0.73–0.89 g m^−2^ day^−1^), west < 1-km (0.80–0.90 g m^−2^ day^−1^), and 1–2 km southwest (0.67–0.87 g m^−2^ day^−1^) of the Met–Mex factory. The lowest average dust deposition fluxes were observed at 2–3 km southeast (0.06–0.27 g m^−2^ day^−1^) and 3 km northwest (0.12 to 0.26 g m^−2^ day^−1^) of the Met–Mex factory (Fig. S1a). There is a tendency to decrease dust fallout rates downwind. Dust deposition fluxes were significantly higher downwind of the Met–Mex Peñoles, with significant increases observed 1–3 km to the southeast (Fig. S1a, Fig. 2b). Dust fall decreased by 70–90% 3 km west and east of the Met–Mex Peñoles.

### Size and morphological characteristics of metal-rich particles

Figure 2 display microphotography of typical metal-rich particles and metal-rich agglomerates of slag observed in the adjacent urban areas in Torreón, within a radius of < 2 km from the Met–Mex complex. Results from SEM examination indicate that metal-rich particles consist primarily of individual angular grains, spheres, rice grains type, and tiny discrete particles. Additionally, amalgamations of spheres and other metal-rich agglomerates particles were observed. The size distribution of individual and agglomerates particles ranged from ultrafine (< 0.1 μm) to giants (50–70 μm diameter or length). In terms of the number of metal-rich particles, the distribution was as follows: Ultrafine particles (< 0.1 µm) 30–49.2 (median 33.5%), fine particles (0.1–2.5 µm) 25–30.6 (27.8%), respirable particles (2.5–4 µm) 14.7–22.2(15.8%), thoracic particles (4–10 µm) 8.2–16.7 (13.9%), and inhalable particles (> 10 µm) 6.1–19.4(9.1%). In terms of mass, the particle size distribution was as follows: 1.3–4.9 (1.9%), 2.7–8.4 (5.6%), 1.3–12.3 (7.6%), 22.4–51.8 (50.9%), and 23–71.6 (33.9%), respectively. Although ultrafine and fine particles are more abundant than larger sizes in terms of counts, they represent < 15% of the emitted mass, as smaller sizes contribute less to the total mass.

### Elemental abundances in metal-bearing particles within settled urban dust

Analysis using SEM–EDS revealed that most particles in the settled urban dust samples consisted of a diverse range of crustal-related components, including quartz, aluminum silicates, and calcium-rich particles (Table S2). These components primarily originate from resuspended dust. Our focus was on identifying metal-bearing particles containing potentially toxic elements (e.g., Cd, Cu, Pb, Zn) based on the chemical composition analyses. The number of metal-bearing particles containing EPTs increased near the Met–Mex facility, and this trend was consistent across all particle sizes. An exception to this pattern was observed in the case of medium-sized particles (< 4 mm), which exhibited an increase in numbers with distance, reaching a maximum at 2.5 km to the east (Fig. S2). A total of 608 metal-bearing particles, varying in sizes and morphologies, were quantified for their elemental composition (in abundance %) across the 32 selected urban dust samples.

Within these samples, we identified a total of 282 Pb-bearing particles, which can be categorized into 85% individual particles and 15% agglomerates. These Pb-bearing particles exhibit equivalent diameters ranging from < 0.1 to 20 μm. Among them, 138 particles are primarily composed of Pb (59–75% of their composition), while 128 contain Pb inclusions within the Fe-silicate matrix (3–46% of Pb). These Pb-bearing particles were representative of sizes, morphologies, and elemental concentrations found in the settled urban dust collected within a 3 km radius of the Met–Mex complex. The mineral and elemental compositions of these Pb-rich particles are provided in Table S2.

The results of the elemental abundances quantification (in abundance %) in metal-rich particles, obtained through SEM–EDS, are summarized in Table 1. A higher prevalence of Pb-rich particles was observed in the urban dust collected near the Met–Mex facility. Peaks corresponding to Pb in EDS spectra were clearly distinguished from other metals (such as Al, Ag, Ca, Cd, Cu, Fe, Hg, Mg, and Zn), metalloids (As, Bi, and Si), and non-metals (Cl, P, and S), which appeared together in the lower part of the spectra. The content of Pb in the selected Pb-particles varied from non-detectable (ND) to 87.2%, with a median value of 54.1%. In addition to Pb, Pb-rich particles comprised 6.4% of Zn, 3.3% Fe, 6% Cu, 2.7% for Ca, 1.5% Cd, 0.9% for Mg, 0.7% Al, 0.3% Ag, and 0.2% of Ti and Hg. Metalloids present in Pb-rich particles included Bi (median 2.1%), Si (2%), and As (1%). Non-metal contents showed median values of 3.4% for S and 0.6% for P. Metal-rich particles observed included Fe, Sn and Zn-rich particles. For instances, Sn-rich and Zn-rich particles showed contents of up to 82 and 90.3% of Sn and Zn, respectively (Table S2).

**Table 1:** Elemental composition of most representative metal-rich particles (% in abundance) in urban dust collected in Torreón (northern Mexico).

Element	Distant urban area from Met–Mex			Urban environmental samples in the vicinity of the Met–Mex Peñoles Site#(distance km − direction)														
	NE 7 km	NE 3 km	NE 5 km	S 1.2 km	SE 0.7 km	E 2.5 km	S 1.7 km	SE 0.7 km	SW 1.8	NNE 1.1 km	NNE 0.7 km	N 1.5 km	NW 0.5 km	NW 1.5 km	NW 3 km	N 0.7 km	NE 2 km	SW 1.1
Size class (μm)				< 4	< 4	> 10	> 10	< 2.5	< 2.5	> 10	< 0.1	> 10	< 2.5	< 0.1	> 10	< 2.5	< 10	< 10
Ag	0.3	ND	0	ND	0.2	0.03	0.8	0.2	ND	0.1	ND	ND	ND	1.3	0.5	1.9	0.1	0.6
Al	12.7	17.2	0	ND	2.1	0.0	1.0	1.4	ND	0.01	1.0	10.3	0.05	0.7	0.6	0.0	2.2	1.2
As	2.5	1.5	0	1.4	1.7	ND	1.3	1.0	ND	ND	ND	3.4	0.04	0.5	0.8	0.2	0.8	1.3
Bi	ND	ND	1.6	ND	ND	0.03	ND	7.2	2.1	ND	7.2	ND	2.5	ND	ND	ND	ND	0.0
Ca	15.9	10.4	72.2	2.3	5.7	0.3	1.6	2.7	2.6	0.8	2.8	ND	ND	ND	ND	ND	8	8.7
Cd	ND	ND	ND	5.1	1.5	0.1	0.4	2.2	ND	ND	ND	2.8	ND	ND	0.2	1.3	ND	3.3
Cl	ND	0.1	ND	ND	ND	ND	ND	ND	ND	ND	ND	ND	ND	ND	ND	ND	2.7	ND
Cu	0.63	0.51	0.77	5.1	6.2	4.9	5.7	5.8	6.1	4.1	6.0	6.9	ND	ND	ND	14.1	5.6	9.5
Fe	6.0	13.9	9.1	0.6	4.1	3.3	63.6	2.9	0.4	5.3	0.4	9.3	0.4	ND	12.3	2.2	59	ND
Hg	ND	ND	0	ND	ND	0.05	ND	ND	ND	ND	0.2	ND	0.6	0.3	0.0	0.3	ND	ND
Mg	ND	0.6	ND	ND	ND	ND	ND	ND	1.5	0.3	ND	ND	ND	ND	ND	ND	ND	ND
P	0.6	0.7	ND	2.5	1.9	0.1	0.1	1.0	0.2	0.1	ND	ND	ND	ND	ND	ND	3	9.8
Pb	ND	0.7	ND	42.4	62.5	0.0	17.4	51.0	73.2	0.3	70.2	7.8	87.2	86.0	1.7	65.6	2.6	57.1
S	1.1	ND	1.7	3.6	2.9	0.03	3.4	4.9	1.2	0.4	8.6	5.4	8.7	9.6	2.5	10.9	0.8	0.7
Si	52.9	48.6	1.2	1.5	6.9	0.9	2.9	16.5	1.6	0.7	2.2	45.0	0.6	1.6	2.5	2.0	15.2	5.0
Ti	1.7	1.0	ND	ND	ND	ND	ND	ND	0.2	0.01	0.4	ND	ND	ND	ND	ND	0.01	ND
Zn	ND	ND	6.4	35.4	4.3	90.3	1.7	3.1	10.9	87.8	1.10	9.2	ND	ND	79.0	1.4	ND	2.8

ND = non-detected or below detection limit (0.05–0.1%).

Table 1 also summarizes the elemental composition of composite samples collected from urban areas located at 4–7 km north-northeast of Met–Mex, representing the urban background in Torreón. The median contents of elements in the distant Met–Mex samples were as follows: Ag 0.2%, Al 12.7%, As 1.5%, Ca 10.4%, Cl 1.4%, Cu 0.56%, Fe 13.9%, Mg 0.6%, P 0.7%, Pb 1.7%, S 1%, Si 48.6%, Ti 1%. No detectable amounts of Bi, Cd and Zn were found in these samples. These results indicate that the composition of the distant Met–Mex samples is a reliable representation of the background urban environment in the region.

**Figure 3:**
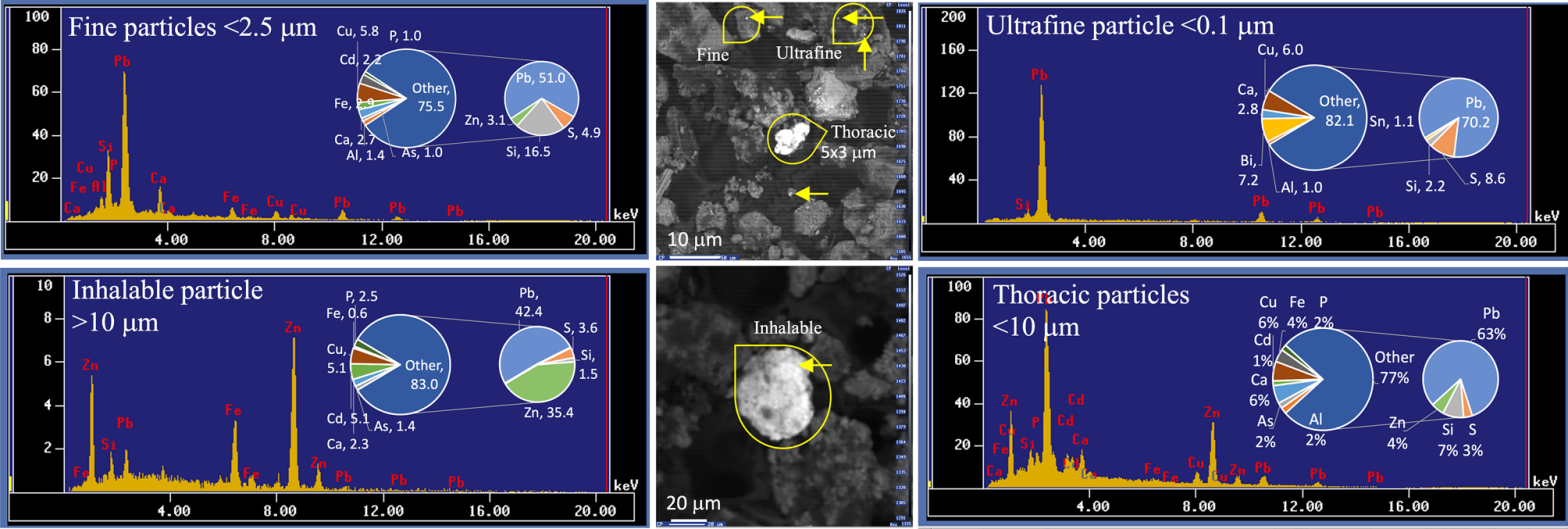
Energy Dispersive X-ray Spectroscopy spectrum in conjunction with scanning electron microscopy and elemental composition (% weight) of Pb-rich particles in urban dust collected in adjacent site to the smelter (TP1-3).

**Figure 4:**
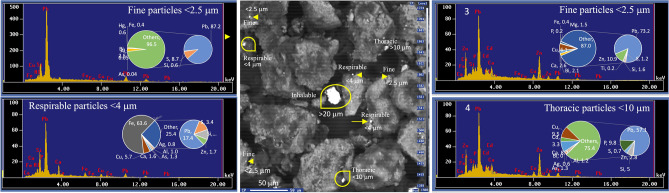
Energy Dispersive X-ray Spectroscopy spectrum in conjunction with scanning electron microscopy and elemental composition (% weight) of Pb-rich particles in urban dust collected in adjacent site to the smelter (TP2-3).

The elemental composition of the particles exhibited significant variation across different observed sizes and morphologies. However, Pb-rich particles with similar morphological characteristics, whether within the same sample or different samples, displayed similarity in their elemental composition. Notably, a relationship between particle size and elemental composition, particularly in terms of Pb content, was observed (Table 1, Figs. 3 and 4). The data reveals that ultrafine (< 0.1 μm) and fine (0.1–2.5 μm) particles showed high Pb contents, ranging from 70.2 to 86% and 51–87.2%, respectively. Similarly, medium-size particles (2.5–4 μm: 42.4–62.5%; 4–10 μm: non-detected to 57.1%) also exhibited elevated Pb contents and a lower proportion of other elements. In contrast, larger metal-rich particles (> 10 μm) accounted for less than 20% of the total Pb mass.

### Identification of minerals in metal-rich particles

To identify the minerals, present in the complex chemical composition of metal-rich particles, which encompass metals such as Al, Ag, Ca, Cd, Cu, Fe, Hg, Mg, and Zn, metalloids such as As, Bi, and Si, and non-metals including P and S, representative samples of urban dust were subjected to X-ray diffraction analyses. Table 2 summarizes the XRD results, focusing on the most prevalent Pb-rich particles. The XRD patterns revealed the presence of galena and sphalerite in the urban dust samples collected near the Met–Mex facility. This was evident from the observed Pb and Zn sulfur peaks in the XRD patterns. The detection of these minerals indicates the emission of sulfide mineral concentrates, which are processed in Met–Mex to produce metallic Pb and Zn ingots. Additionally, remnants of galena were observed in the expelled slags (> 10 μm), providing evidence of incomplete smelting of the processed concentrates.

**Table 2:** Major and minor mineral phases of Pb identified in metal-rich particles in urban dust samples collected in the Pb-polluted urban environment area from Torreón (northern Mexico). Mineral phases are alphabetically ordered.

Pb phase	Formulation	Selected weathered Pb-rich particles										
		S 1.2	SE < 1	E 2.5	W < 3	W < 1	SW < 2	NNE < 1	NW 3	NE 2	NW 0.5	SW 1.1
Major minerals												
Anglesite	PbSO_4_		+	+				+	+	+?	+	+
Cerussite	PbCO_3_		+	+	+	+	+	+			+	+
Galena	PbS	+	+	+		+	+	+		+	+	+?
Hydrocerussite	Pb_3_(CO_3_)_2_(OH)_2_	−	+	+?	+	+	+	+			+?	+?
Hydroxylpyromorphite	Pb_5_(PO_4_)_3_(OH)	+	+?	+?	+							+?
Litharge	PbO		+			+						+
Minor minerals												
Caledonite	Pb_5_Cu_2_(SO_4_)_3_(CO_3_)(OH)_6_	+	+	+	+			+			+	+
Crimsonite	PbFe_2_(PO_4_)_2_(OH)_2_		+	+				+				+
Esperite	PbCa_2_Zn_3_(SiO_4_)_3_	+	+?	+?	+	+						+
Feinglosite	Pb_2_Zn(AsO_4_)_2_	+	+	+								+
Friedrichite	Pb_5_Cu_5_Bi_7_S_18_					+	+		+			+?
Galenobismutite	PbBi_2_S_4_					+	+		+			
Lautenthalite	PbCu_4_(SO_4_)_2_(OH)_6_	+	+	+	+						+	+
Linarite	PbCu(SO_4_)(OH)_2_	+	+	+	+						+	
Luddenite	Cu_2_Pb_2_Si_5_O_14_		+					+			+	
Massicot	PbO		+?			+?						+?
Vanackerite	Pb_4_Cd(AsO_4_)_3_(Cl,OH)	+	+		+							+

+ = present, +? = seems present, empty = non-observed.

**Figure 5:**
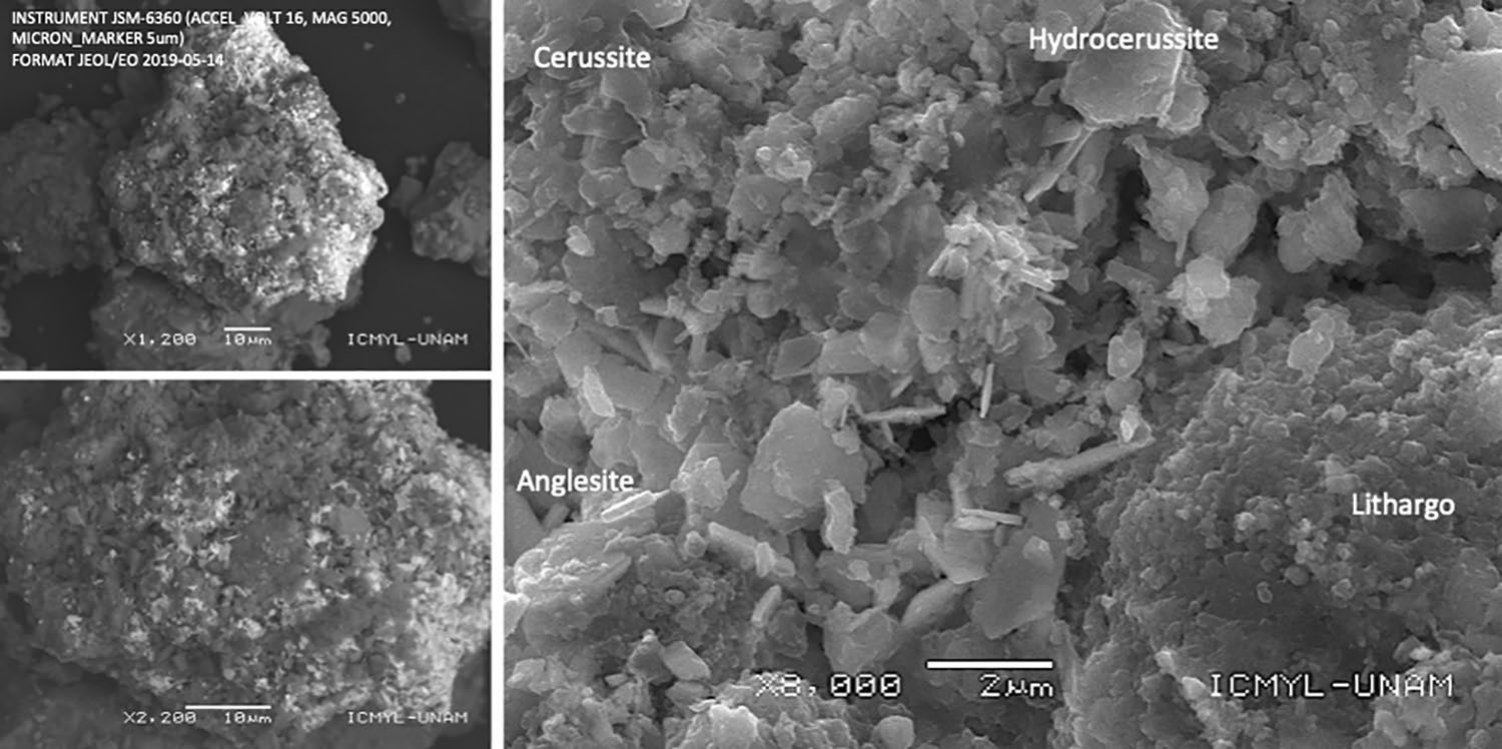
Surface morphology and secondary phases onto weathered Pb-rich particles in urban dust collected in the vicinity of Met–Mex as observed by SEM. The presence of Pb carbonate minerals (cerussite, hydrocerussite), Pb-sulfate (anglesite), and Pb-oxides (litharge) were identified in weathered Pb-rich particles.

The analysis of Pb-rich individual particles and galena-containing slags revealed the presence of secondary Pb minerals, primarily the Pb carbonates such cerussite (PbCO_3_) and possibly hydrocerussite (Pb_3_(CO)_2_(OH)_2_) (Fig. 5; Table 2). Cerussite, characterized by pseudo-hexagonal form crystals, appeared as thin, snowflake-like masses on the Pb-rich particles. Similarly, hexagonal hydrocerussite crystals were also observed on the Pb-rich particles. The weathered galena particles were notably covered with cerussite.

Additionally, the data indicated the presence of other secondary Pb minerals within the Pb-rich particles, such as litharge and/or massicot (PbO), hydroxyl-pyromorphite (Pb_5_(PO_4_)_3_(OH)), and anglesite (PbSO_4_). A list of minor secondary Pb minerals was also observed in the urban dust collected in the Met–Mex vicinity. However, further investigations are required to differentiate the structures of these minerals.

## Discussions

Elevated dustfall rates were recorded in the urban areas of Torreón, an arid city, with the highest values observed within a 1–2 km radius surrounding one of the world's largest Ag–Cd–Pb–Zn smelting and refining complexes, Met–Mex Peñoles, which has been in operation since 1901. During the months from November to March, thermal inversions and powerful convection flows result from the interaction between cold compressed air and warm air, leading to the resuspension and mobilization of particles into the atmosphere. This resuspension phenomenon can even trigger dust storms, locally referred to as 'tolvanera', which emit substantial amounts of dust into the atmosphere, potentially dispersing over long distances.

Over several decades, Met–Mex released significant quantities of dust into the Torreón atmosphere, stemming from uncontrolled processes without filtration (1900s–1960s) and processes with limited emission controls (1960s–1990s)^6^. The total inventory of lead (Pb) emissions from the complex amounted to 23,350–27,580 tons, with the majority (63–75%) released during periods of negligible emission controls before 1960. At its peak, the Torreón metallurgical complex released 240 tons per year. Subsequently, emission factors gradually decreased to 65–109 tons per year in the 1990s, with significant enhancements in dust control efficiency from 1999 to 2001 to the present, reducing emissions to 5.4–21.5 tons per year^6^. Dust emissions comprised droplets of slag and matte, condensed particles, and minerals that did not reach the required melting temperature during smelting and refining processes, as well as fugitive emissions of galena and sphalerite ores from various process steps and different sections of the plant.

### Morphologies and size distribution of metal-rich particles

In our study, representative metal-bearing particles of Pb–Zn smelter emissions found in settled urban dust were identified, characterized, and analyzed for mineral identification by using a combination of conventional techniques such as scanning electron microscopy (SEM), Energy Dispersive X-ray Spectroscopy (EDS), and X-ray powder diffraction (XRD). Regarding the morphologies and size distribution of metal-rich particles found in the settled dust samples collected in the urban environment in Torreón, our analysis revealed the presence of two primary categories: spherically shaped particles and angular or irregularly shaped particles, often forming agglomerates of small spherical particles.

The spherical shape of Pb-rich and agglomerate-bearing particles indicates a high-temperature process, such as smelting. These Pb-rich particles are formed by quenched melt droplets during smelting of Pb and Zn concentrate ores, and flue gas cleaning processes emitted by the smelter smokestack^7,10,12,37–42^. On the other hand, angular particles are typically associated with fugitive emissions of dust particles from concentrate ores and other raw materials during handling operations (e.g., transportation, storage, or use) and expelled materials not heated to the melting temperature^7,41,42^.

In typical pyrometallurgical Pb smelters equipped with dust filters, most particles are around 5 µm in size (90%), with the remaining in the 10–20 µm size range^31^. Smelter operations lacking dust filters commonly result in the presence of metal-rich particles and metal-rich slag agglomerates > 20 µm^43^. Thus, the release of smaller particles from Met–Mex primarily occurs during smelting and refining operations, while the emission of larger particles, including agglomerates, may result from fugitive concentrate ore emissions during handling operations and incomplete smelter operations.

The Torreón complex has been effectively capturing high percentages of SO_2_ (> 99.5%) and particulate matter (> 99.5%) using acidic plants and electrostatic precipitators for over two decades^6^. Therefore, we assumed that the metal-bearing matter found in the settled urban dust in the Torreón urban area is more likely to be resuspended rather than freshly emitted by Met–Mex.

It is expected that coarser particles are deposited in the vicinity of the factory and the surrounding urban environments, while fine and ultrafine particles can travel longer distances away from the complex^43–46^. In our study, the number of metal-bearing particles of different sizes decreased with proximity to the Met–Mex facility. An exception was observed for medium-sized particles (< 4 μm) that increased with Met–Mex distance, peaking at 2.5 km to the east. The limited study area (< 3 km radius) could explain why the tiny particles did not decrease with distance.

In our study, metal-rich particles mixed in the urban dust accounts < 10% for PM_2.5_ and close to 60% for PM_10_, while ultrafine (< 0.1 μm) and fine (0.1–2.5 μm) close 10%, and > 30% for inhalable particles > 10 μm. These fine metal-rich particles represent an elevated health risk for the population regarding to the tiny particles but mainly because the elevated contain significant levels of toxic metals, including Pb, Cd, and Zn. For example, our analysis by SEM–EDS techniques of collected PM samples revealed that the content of Pb-rich particles ranging from 60 to 70% in PM_10_ and PM_2.5_.

The particle size distribution is an important parameter for determining particle dynamics, displacement, deposition, and the duration of metal-rich particle suspended in the atmosphere. The size of the metallic particles is also prime for human incorporation. The finest size metallic particles are more easily inhaled, ingested, and absorbed through dermal contact. For example, fine particles (< 2.5 μm) can penetrate the smaller airways in the peripheral lung region close to alveolar interstitium, while ultrafine particles (< 0.1 μm) can penetrate until the alveolar interstitium. In the alveolar region of the lungs, the rate of absorption of Pb particles is about 32% of the deposition in the lungs^47^. Additionally, the finest particles adsorb more heavy metals compared to coarse-grained ones, which is attributed to the larger surface areas of their components^17^.

### Mineral composition of weathered Pb-rich particles

No previous studies have described the mineralogy of metal-rich particles emitted by the Ag–Cd–Pb–Zn smelter and refining complex in Torreón. Pb rich-particles emitted from non-ferrous smelter and refining complexes contain a variety of compounds, including sulfides (PbS, ZnS) and their oxidation products such as sulfates (PbSO_4_), oxides (PbO, ZnO, CdO, As_2_O_3_), oxide-sulfates (PbO⋅PbSO_4_), carbonates (PbCO_3_), Cl-bearing phases (PbClOH, Pb_4_O_3_Cl_2_), metallic elements (Pb^0^, Zn^0^) and Pb silicates^32,34,42^.

All these minerals were observed in our collected dust samples. Secondary minerals were mostly observed around or in incrustations within cavities of primary minerals, such as galena, and in molten agglomerates. A notable absence of Cl-bearing phases was observed in our urban dust. This can be explained by the difficulty in identifying non-crystalline phases by XRD and/or their high solubility, which causes rapid dissolution and mobilization into the environmental matrices (from aerosols to urban dust soils, to soils and water)^48^.

Although XRD is a valuable method for mineral identification, additional physical or chemical tests may be necessary to distinguish minerals with similar crystalline structure. Further investigations using complementary techniques, such as Raman spectroscopy^15^, will contribute to a more comprehensive characterization of these minerals in the urban dust near the Met–Mex facility.

Although the formation of secondary Pb and Zn minerals during the pyrometallurgical process, as transformation products, is highly probable, the chemical composition of Pb-particles may change over time during weathering^23,31,49,50^.

The presence of secondary Pb minerals can also be associated to the ore concentrate coming from the mines to feed the Met–Mex smelter. In Mexico, the production of silver, cadmium, lead, and zinc primarily relies on the exploitation sphalerite (ZnS) and galena (PbS). Galena and sphalerite may also be accompanied by a minor presence of other secondary minerals, including cerussite (PbCO_3_), smithsonite (ZnCO_3_), smithsonite (ZnCO_3_), maghemite (FeZnCO_3_), hydrozincite (Zn[(OH)_3_CO_3_]_2_), and hemimorphite (Zn_2_SiO_4_). Notably, these secondary minerals form through the weathering of the lead and zinc sulfides and are primarily found in shallow deposits. We assumed that the presence of secondary minerals in the concentrates processed by Met–Mex is highly improbable because Peñoles and its subsidiaries operate both underground and open pit mines, typically extracting ores from depths of up to 1000 m below surface outcrops. Furthermore, the flotation and lixiviation processes employed during the concentration of galena and sphalerite effectively remove impurities, including probably these secondary minerals. Even if some secondary Pb minerals were to arrive in the smelter feed concentrate, the carbonate minerals easily break down during Pb oxidation at high temperatures. Thus, the presence of first-generation secondary Pb minerals is negligible, such as was observed in the analyzed samples. Besides, the metal-bearing particles analyzed in our study are presumed to be resuspended rather than freshly emitted by Met–Mex.

The presence of secondary minerals should be indicative of the degree of oxidation before the subsequent processing (smelting, refinery) and alteration during and after emission to the atmosphere, deposition, and accumulation in the urban area. The formation of secondary phases and the release of potentially toxic elements during the weathering of metal-bearing particles have been extensively investigated (e.g.,^48–52^). Following the insights of these authors, the weathering process of Pb-rich particles that may contain PbS inclusions or consist of PbS (galena), begins with the gradual oxidation of sulfides into sulfates (step 1). Then galena is converted to anglesite. Subsequently, Pb(SO_4_)_2_ dissolves, liberating Pb^2+^ and (SO_4_)^2−^ from the particle’s interior or surface into the pore water (step 2). In carbonated soils, such as those in Torreon (> 20% in weight), Pb^2+^ reacts with elevated levels of (CO_3_)^2−^, leading to the formation of Pb-bearing carbonates (step 3a). The formation of cerussite (PbCO_3_) and/or hydrocerussite (Pb_3_(CO_3_)_2_(OH)_2_) occurs. Alternatively, Pb^2+^ may precipitate as a hydroxide near the inclusion, which can undergo transformation into Pb-bearing oxides (step 3b), including litharge. This process culminates, over time, in the creation of a weathering crust around Pb-bearing sulfide particles, involving sulfates, carbonates, and oxide formations.

In this study, the presence of cerussite, litharge, and anglesite around pieces of galena may indicate that PbS has undergone partial oxidation^16^. Numerous particles of galena and Pb-bearing particles with PbS inclusions, have been converted into secondary minerals of Pb, providing clear evidence of weathering processes affecting these sulfide mineral particles. The presence of these secondary Pb minerals, as coatings on altered Pb-bearing particles, was illustrated in Fig. 5.

Nevertheless, the presence of particles of galena as the primary phase in urban dust also suggest a slow kinetic weathering process^15–17^, giving that emissions began over 120 years ago and that most Pb-particles were emitted prior to the 1960s^6^. Changes in the mineralogy of the Pb-rich particles historically emitted by Pb–Zn smelting and refining activities evidence the progress in weathering. Pb-bearing carbonates and then sulfates form were the predominant alteration products of Pb-sulfide mineral found around pre-existing sulfide inclusions of galena pieces, and in lesser extent Pb-bearing oxides. The implementation of a geochemical fractionation protocol is required to determinate the phase partitioning of Pb in the settled urban dust.

### Environmental impacts of lead

Upon emission, galena (PbS) comes into contact with environmental elements such as air and water, which accelerate its weathering and facilitate the subsequent development of lead phases, such as PbSO_4_ and PbCO_3_^50–52^. This weathering process transforms galena into lead phases characterized by higher Pb contents and enhanced bioaccessibility compared to the original galena. For instance, cerussite and anglesite exhibit Pb contents of up to 77.5% and 74%, respectively, surpassing the 50–60% Pb content of galena. Importantly, these lead phases demonstrate increased bioaccessibility compared to galena^53^. Under gastric conditions, lead carbonates and lead sulfates are notably more soluble than galena^34,53–57^. In addition to chemical transformations, the reduction in the size of metal-bearing particles results in an increase in their surface area. A larger surface area renders the particles more prone to dissolution, consequently enhancing the bioavailability of metals. To assess the human health risk, it is advisable to perform extraction tests using simulated body fluids (e.g., gastric and alveolar lung fluids) in environmental matrices within Torreón. These tests are valuable predictors of the mobility and bioaccessibility of Pb and other EPTs.

Thus, the weathering alterations of Pb-rich particles to finer particles, more concentrated in Pb, and forming secondary minerals exacerbates the solubility, availability, and toxicity of Pb in urban environments^31,34^. The Pb-rich particles undergoing weathering are susceptible to be more accessible and available when they are incorporated into the human body through different routes, including inhalation in aerosols, ingestion in soils and dust, and absorption by dermal contact^58^.

This study presents compelling evidence of the physicochemical transformation of lead (Pb)-rich particles, which is manifested through alterations in their particle size, elemental composition, and mineral content. Specifically, secondary minerals become incorporated into significantly smaller particles, mostly ranging from < 0.1 to 4 μm in size, in contrast to the larger original sulfide particles (> 20 μm).

Given the enduring presence of Pb and its ongoing physical and chemical changes, it is imperative that Met–Mex takes proactive measures by implementing an extensive remediation plan, covering a wide radius around the Torreón complex. The remediation efforts should encompass the removal of significant quantities of Pb and other potentially toxic elements that were released into the urban environment of Torreón over more than one century and are being mobilized in urban dust. Otherwise, the pollution will persist for decades and adversely impact the surrounding population. These efforts also involve aiding in mitigating the adverse effects on the affected people. Currently, there is not even a complete registry of those affected and the health consequences they are suffering from chronic exposure to metals. Furthermore, there is a pressing need for Met–Mex to implement stringent emissions controls at the smelting-refining process, incorporating state-of-the-art filtration technology at each stage of their processes, and to prevent more emissions of metal-rich particles within urban environments. Failure to do so may necessitate the consideration of closing the Pb-smelting operations within the Met–Mex complex, which is situated in the heart of Torreón city and installing a state-of-the-art metallurgical complex in an industrial zone.

In addition to these measures, it is crucial that Met–Mex allocates resources to support independent scientific studies focused on assessing the risks posed to the population of Torreón. The urban area surrounding Met–Mex spans 21.2 km^2^, a 14% of the Torreón urban area of 152.89 km^2^. Drawing from a population density of 6401 people km^2^^59^, the population within this affected region is of 135,740 people^24^. Pb-rich particles can enter the human body through inhalation and ingestion pathways, and a portion of the absorbed Pb is transported and accumulates in tissues, resulting in harm to bones, the brain, liver, kidneys, and causing microcytic anemia^60,61^. Considering the age distribution of the population, we have estimated that there are 33,500 individuals under the age of 14 years residing in this area. This figure encompasses 11,100 children aged between 0 and 4 years and an additional 11,200 children aged between 5 and 9 years^24^. In this vulnerable sector of the population, even low blood lead levels (< 2 μg dL^−1^) can have detrimental effects on IQ, attention span, and academic performance^58,59^.

## Conclusions

In Torreón, an arid city hosting one of the world's largest Ag–Cd–Pb–Zn smelting and refining complexes, Met–Mex Peñoles, dustfall rates are notably high, particularly within a 1–2 km radius around the complex (0.67–0.90 g m^−2^ day^−1^). These dustfall rate (DFR) values are considered maximum due to the timing of our study, conducted during seasons characterized by thermal inversions and convection flows. These meteorological conditions result in the resuspension and mobilization of substantial volumes of urban dust into the atmosphere, including a greater number of metal-bearing particles.

We conducted a comprehensive analysis of representative metal-bearing particles from Pb–Zn smelter emissions found in settled urban dust using various techniques, including scanning electron microscopy (SEM), Energy Dispersive X-ray Spectroscopy (EDS), and X-ray powder diffraction (XRD). Currently, significant amounts of metal-rich particles, including Pb-rich particles, occur in the urban dust surrounding Met–Mex in Torreón. These particles exhibit a variety of sizes, morphologies, and elemental and mineral compositions. Metal-bearing particles showed varied morphology (spherically shaped particles and angular or irregularly shaped particles and forming agglomerates of small spherical particles) and sizes (from < 0.1 to > 10 μm, with predominance of < 4 μm), which is related to Pb–Zn smelter emissions.

About a third part of the metal-bearing particles containing EPTs found were Pb-rich particles. In Torreón, Pb-rich particles that were historically emitted for over one century continue in the modern urban dust. Lead is a persistent pollutant with limited mobility, which means that the pollution will continue for centuries unless major abatement operations are employed.

Analyses of particle surface structure by SEM, and elemental and mineral composition using SEM–EDS and XRD techniques reveal that weathering processes in being altering the Pb-rich particles. The weathering increases the presence of secondary minerals such as Pb carbonates, Pb sulfate, and Pb oxides, which contain higher amounts of Pb than the primary sulfides. Additionally, the weathering of Pb-rich particles is causing their particle sizes to decrease, resulting in finer particles. Pb-rich particles can enter the human body through inhalation and ingestion pathways, with a portion of the absorbed Pb being transported to target organs, leading to multiple health effects. Among the most vulnerable population segments are infants, with 11,080 children aged 0–4 years and 11,190 children aged 5–9 years residing in the affected area. Epidemiological studies should investigate the potential hazards associated with Pb-rich particles emitted from the smelter complex.

The physical–chemical transformations of weathered Pb-rich particles are enhancing the solubility and availability of Pb in Torreón's urban environment, thereby exacerbating its toxicity and its health impact on the population. Understanding the long-term weathering alterations of metal-rich particles emitted from non-ferrous smelting and refining operations is crucial for comprehending the mechanisms responsible for the release of Pb and other potentially toxic elements (PTEs) into the environment. To gain a deeper understanding of the natural weathering process of metal-bearing particles from primary Pb–Zn smelting in Torreón, the application of Raman microspectroscopy is recommended. Furthermore, implementing a geochemical fractionation protocol and conducting extraction tests using simulated body fluids (e.g., gastric and alveolar lung fluids) is essential to determine the phase partitioning of Pb in settled urban dust and assess potential human health risks in the Torreón population.

Overall, it is evident that the pollution caused by Pb-rich particles in Torreón is a persistent problem that requires immediate action to prevent long-term consequences on the environment and human health.

### Supplementary Information

Supplementary Information.
