# Supplementary material for: Environmental and health implications of Pb-bearing particles in settled urban dust from an arid city affected by Pb–Zn factory emissions
Source: Sci Rep. 2023 Dec 2;13:21287. doi: 10.1038/s41598-023-48593-5 (PMC10693616; doi:10.1038/s41598-023-48593-5)
Supplement: Supplementary file 1 — Supplementary Information. [file 41598_2023_48593_MOESM1_ESM.docx]

Supplementary material

**Environmental and health implications of Pb-bearing particles in settled urban dust from an arid city affected by Pb-Zn factory emissions**

M.F. Soto-Jiménez^a*^, S. Roos-Muñoz^a,b^, S. Soto-Morales^c^, L.E. Gómez-Lizarrága^d^, L. Bucio-Galindo^e^

^a^Unidad Académica Mazatlán, Instituto de Ciencias del Mar y Limnología, Universidad Nacional Autónoma de México. Mazatlán, Sinaloa, México.

^b^Tecnológico Nacional de México/Instituto Tecnológico de Mazatlán, Mazatlán, Sinaloa, México.

^c^Instituto Nacional de de Salud Pública, Cuernavaca, Morelos, México.

^d^Instituto de Ciencias del Mar y Limnología, Universidad Nacional Autónoma de México. Ciudad Universitaria, Mexico city.

^e^Laboratorio de Cristalografía y Materiales Naturales, Instituto de Física, Universidad Nacional Autónoma de México. Ciudad Universitaria, Mexico city, México.

Correspondent author:

Telephone number: +52 (669) 9852845 to 48.

Fax number: +52 (669) 9826133

E-mail: [martin@ola.icmyl.unam.mx](mailto:martin@ola.icmyl.unam.mx)

ORCID 0000-0001-7584-3684

Table S1. Variation spatial-temporal of dust fall rates (DFR, g m^-2^ d^-1^) and Pb-load (mg m^-2^ d^-1^) determined by collection of atmospherically deposited material sinking by dry deposition in the urban area from Torreón in 2015 and 2017 surveys.

| Direction and distance to Met-Mex complex (n=5-6 samples per site-month) | Jan | Feb | Mar | Apr | May |
| --- | --- | --- | --- | --- | --- |
|  | Dust fall rate (g m^-2^ d^-1^) | | | | |
| NW 0.5 km | 0.82±0.21 | 0.90±0.08 | 0.80±0.25 | 0.84±0.19 | 0.81±0.05 |
| NW 1 km | 0.67±0.47 | 0.48±0.24 | 0.47±0.36 | 0.30±0.11 | 0.34±0.28 |
| NW 1.5 km | 0.48±0.25 | 0.45±0.14 | 0.55±0.22 | 0.64±0.23 | 0.47±0.22 |
| NW 3 km | 0.23±0.11 | 0.26±0.15 | 0.15±0.23 | 0.22±0.10 | 0.12±0.16 |
| N 0.7 km | 0.53±0.23 | 0.66±0.16 | 0.70±0.09 | 0.56±0.17 | 0.51±0.26 |
| N 1.5 km | 0.25±0.06 | 0.19±0.12 | 0.25±0.24 | 0.21±0.16 | 0.11±0.07 |
| NE 0.7 km | 0.28±0.10 | 0.57±0.30 | 0.57±0.26 | 0.41±0.34 | 0.40±0.48 |
| ENE 2.7 km | 0.26±0.14 | 0.25±0.08 | 0.24±0.09 | 0.23±0.07 | 0.24±0.14 |
| NE 2 km | 0.24±0.13 | 0.29±0.14 | 0.28±0.13 | 0.25±0.08 | 0.23±0.11 |
| NE 3 km | 0.17±0.13 | 0.15±0.06 | 0.24±0.29 | 0.21±0.23 | 0.19±0.10 |
| NE 5 km | 0.26±0.13 | 0.23±0.14 | 0.23±0.11 | 0.23±0.11 | 0.20±0.10 |
| E 1.9 km | 0.31±0.04 | 0.31±0.11 | 0.36±0.16 | 0.33±0.07 | 0.19±0.07 |
| E 2.5 km | 0.27±0.23 | 0.06±0.05 | 0.15±0.13 | 0.12±0.09 | 0.10±0.06 |
| SE 0.7 km | 0.61±0.32 | 0.67±0.24 | 0.68±0.26 | 0.65±0.34 | 0.47±0.21 |
| SE 2.7 km | 0.29±0.13 | 0.25±0.09 | 0.27±0.10 | 0.23±0.08 | 0.22±0.06 |
| S 1.2 km | 0.44±0.13 | 0.35±0.10 | 0.37±0.14 | 0.26±0.05 | 0.25±0.04 |
| S <2km | 0.27±0.12 | 0.51±0.26 | 0.46±0.41 | 0.24±0.08 | 0.35±0.37 |
| SW 1.1 km | 0.78±0.24 | 0.86±0.26 | 0.73±0.21 | 0.77±0.24 | 0.89±0.13 |
| SW 2 km | 0.81±0.21 | 0.83±0.23 | 0.67±0.23 | 0.72±0.24 | 0.87±0.12 |
| W 2 km | 0.56±0.21 | 0.64±0.15 | 0.70±0.08 | 0.59±0.16 | 0.55±0.25 |
| W 2.7 km | 0.16±0.06 | 0.16±0.04 | 0.15±0.08 | 0.15±0.04 | 0.13±0.05 |

Table S2. Type of mineral and elemental composition evaluated by SEM-EDS in metal-rich particles collected in urban dust fallout collected in 3 km-radius in all directions around the Met-Mex complex (2015-2017 surveys).

| Mineral type | Major elements  (min-max wt. %) | Counts per 1000 particles | Identified  particles | Abundance |
| --- | --- | --- | --- | --- |
| Si-rich | Si-dominant (56-87) | 419(408-530) | 19,215 | 47.2% |
| Si+Al | Si(55-66); Al(5-14) | 372(224-386) | 12,015 | 29.5% |
| Si+Fe | Si(26-41); Fe(11-53) | 163(110-166) | 5,483 | 13.5% |
| Si+ Ca | Si(11-45); Ca(5-17) | 31(6-122) | 3,004 | 7.4% |
| Si+Cu+Zn | Si(41); Cu(6-9); Zn(7-11) | 3(2-4) | 119 | 0.29% |
| Si+Pb+Zn | Si(46-68); Pb(3-36); Zn (3-12) | 1.8(0.2-2) | 40 | 0.10% |
| Si+Cu | Si(16-60); Cu(4-5) | 1.0(0.2-1.1) | 24 | 0.06% |
| Si+P+Al | Si(50-57); P(5-25); Al(6-8) | 1.0(0.2-1.1) | 24 | 0.06% |
| Si+P | Si(8-57);P(5-25) | 0.2(0.1-0.3) | 8 | 0.02% |
| Fe-rich | Fe-dominant (63-97) | 1.0(0.2-1.1) | 24 | 0.06% |
| Fe+Si | Fe(14-79); Si(13-23) | 4.1(3.9-7.5) | 199 | 0.49% |
| Fe+Pb+Si | Fe(60-63); Pb(18-20); Si (4-5) | 3.1(0.2-3.2) | 64 | 0.16% |
| Fe+Pb+Cu | Fe(25-75); Pb(4-5); Cu(3) | 0.2(0.1-0.3) | 8 | 0.02% |
| Fe+Ca+Si | Fe(33); Ca(23); Si (20) | 0.2(0.1-0.3) | 8 | 0.02% |
| Ca-rich | Ca-dominant (73-79) | 0.2(0.1-0.3) | 8 | 0.02% |
| Ca+Si | Ca(5-24); Si(4-16) | 1.1(1.0-1.4) | 48 | 0.12% |
| Ca+Cu | Ca(28-55); Cu(1-4) | 0.2(0.1-0.3) | 8 | 0.02% |
| Ca+Mg | Ca(4-22); Mg(2-9) | 0.2(0.1-0.3) | 8 | 0.02% |
| Ca+P | Ca(9-21); P(1-19) | 0.3(0.1-0.3) | 8 | 0.02% |
| Pb-rich | Pb-dominant (59-75) | 1.5(1.3-1.8) | 138 | 0.34% |
| Pb-Si | Pb(12-38); Si(8-25) | 0.7(0-1.1) | 24 | 0.06% |
| Pb-P-Si | Pb(46); P(17); Si(8) | 0.2(0.1-0.3) | 8 | 0.02% |
| Sn-rich | Sn-dominant (78-82) | 0.8(0.6-1.1) | 32 | 0.08% |
| Zn-rich | Zn-dominant (76-90) | 0.5(0.5-0.7) | 143 | 0.35% |
| Others | Different metals | 1.3(1.2-1.8) | 32 | 0.08% |

Figure S1. a) Variation of the dust fall rates (DFR, g m^-2^ d^-1^) in function of the distance to the Met-Mex Peñoles main stack smelter (arrow yellow), b) Map of Torreón city with the spatial distribution of the averaged DFR by collection of atmospherically deposited material sinking by dry deposition in the urban area from Torreón in 2015 and 2017 surveys. Map modified from EnviroAtlas (1).

Figure S2. Size distribution of metal-bearing particles as a function of the distance from Met-Mex, identified using SEM-EDS in settled urban dust collected in the Torreón area during the 2015 and 2017 surveys.

**References**

1. U.S. Environmental Protection Agency. EnviroAtlas. Torreón — Coahuila de Zaragoza, México. Retrieved: May 31, 2023. <https://enviroatlas.epa.gov>.
